# {2-[2-(Carb­oxy­meth­oxy)­phen­oxy]acetato}[2,2′-(*o*-phenyl­enedi­oxy)diacetic acid]sodium 4,4′-bipyridine hemisolvate monohydrate

**DOI:** 10.1107/S1600536810024797

**Published:** 2010-07-03

**Authors:** Liang Xu, Li-Yan Gu, Yan-Yun Yang, Bing Wang, Ting-Guo Kang

**Affiliations:** a College of Pharmacy, Liaoning University of Traditional Chinese Medicine, Dalian 116600, People’s Republic of China

## Abstract

In the title compound, [Na(C_10_H_9_O_6_)(C_10_H_10_O_6_)]·0.5C_10_H_8_N_2_·H_2_O, the Na atom is eight-coordinated in an distorted dicapped-octa­hedral geometry by eight O atoms from a 2-(2-carb­oxy­meth­oxy­phen­oxy)acetate (*o*-BDOAH) anion and a 2,2′-(*o*-phenyl­enedi­oxy)diacetic acid (*o*-BDOAH_2_) mol­ecule. The uncoordinated 4,4′-bipyridine mol­ecule is arranged around an inversion center and exhibits rotational disorder. A three-dimensional supra­molecular network is built up in the crystal through O—H⋯O and O—H⋯N hydrogen bonds between the uncoordinated water mol­ecule, the uncoordinated 4,4′-bipyridine mol­ecule and the sodium complex mol­ecule.

## Related literature

For a related structure with *o*-BDOAH_2_ and 4,4′-bipyridine, see: Gao *et al.* (2006 ▶).
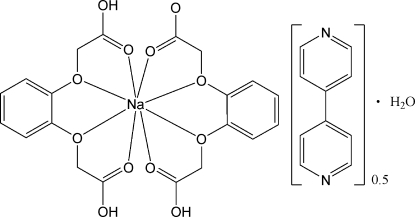


## Experimental

### 

#### Crystal data

[Na(C_10_H_9_O_6_)(C_10_H_10_O_6_)]·0.5C_10_H_8_N_2_·H_2_O
*M* *_r_* = 570.45
Triclinic, 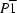

*a* = 8.427 (4) Å
*b* = 13.135 (7) Å
*c* = 13.411 (9) Å
α = 111.08 (2)°
β = 102.03 (2)°
γ = 103.17 (2)°
*V* = 1277.9 (13) Å^3^
*Z* = 2
Mo *K*α radiation
μ = 0.14 mm^−1^
*T* = 291 K
0.24 × 0.23 × 0.21 mm

#### Data collection

Rigaku R-AXIS RAPID diffractometer
Absorption correction: multi-scan (*ABSCOR*; Higashi, 1995 ▶) *T*_min_ = 0.968, *T*_max_ = 0.972
9959 measured reflections
4457 independent reflections
3497 reflections with *I* > 2σ(*I*)
*R*_int_ = 0.022

#### Refinement

*R*[*F* ^2^ > 2σ(*F* ^2^)] = 0.066
*wR*(*F* ^2^) = 0.213
*S* = 1.14
4457 reflections
392 parameters
152 restraints
H-atom parameters constrained
Δρ_max_ = 0.79 e Å^−3^
Δρ_min_ = −0.43 e Å^−3^

### 

Data collection: *RAPID-AUTO* (Rigaku, 1998 ▶); cell refinement: *RAPID-AUTO*; data reduction: *CrystalClear* (Rigaku/MSC, 2002 ▶); program(s) used to solve structure: *SHELXS97* (Sheldrick, 2008 ▶); program(s) used to refine structure: *SHELXL97* (Sheldrick, 2008 ▶); molecular graphics: *SHELXTL* (Sheldrick, 2008 ▶); software used to prepare material for publication: *SHELXL97*.

## Supplementary Material

Crystal structure: contains datablocks I, global. DOI: 10.1107/S1600536810024797/si2266sup1.cif

Structure factors: contains datablocks I. DOI: 10.1107/S1600536810024797/si2266Isup2.hkl

Additional supplementary materials:  crystallographic information; 3D view; checkCIF report

## Figures and Tables

**Table 1:** Hydrogen-bond geometry (Å, °)

*D*—H⋯*A*	*D*—H	H⋯*A*	*D*⋯*A*	*D*—H⋯*A*
O1—H62⋯O8^i^	0.83	1.70	2.517 (4)	173
O6—H61⋯N1*B*	0.82	1.76	2.568 (7)	168
O6—H61⋯N1*A*	0.82	1.76	2.573 (5)	171
O13—H132⋯O2^ii^	0.85	2.16	2.958 (5)	156

Symmetry codes: (i) 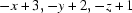; (ii) 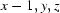.

**Fig. 1.**
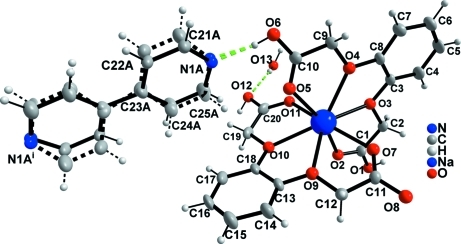
The molecular structure of the title compound, showing displacement ellipsoids at the 30% probability level for non-H atoms. Dashed lines indicate the hydrogen bonds. Symmetry code i: -x + 1, -y, -z. Atoms that belong to the minor fraction of the disordered bipyridine molecule were omitted for clarity.

## supplementary crystallographic information

### Comment

1,2-Phenylenedioxydiacetic acid *o*-BDOAH_2_ is a multidentate flexible ligand, which can be regarded as an excellent candidate for the construction of supramolecular architectures through the coordination or hydrogen bonding interaction (Gao *et al.* 2006). Herein, we report the title compound structure, synthesized by the reaction of *o*-BDOAH_2_, 4,4'-bipyridine and NaOH in an aqueous solution.

The title compound consists of a Na(*o*-BDOAH)(*o*-BDOAH_2_) coordinated complex, a water molecule and hemi-4,4'-bipyridine molecule. The sodium is eight-coordinated in an distorted dicapped octahedral geometry environment defined by eight oxygen atoms from a *o*-BDOAH molecule and a *o*-BDOAH~2~ molecule. The uncoordinated 4,4'-bipyridine molecule is arranged around an inversion center and exhibits a rotational disorder with occupancy of 0.682 (17) and 0.318 (17) for two splitting positions, respectively. (Figure 1, Table 1).

A three-dimensional supramolecular network is built up through O—H···O and O—H···N hydrogen bonds between the lattice water molecule, uncoordinated 4,4'-bipyridine molecule and the sodium complex (Table 2).

### Experimental

*o*-BDOAH_2_ was prepared by the reaction of chloroacetic acid with *o*-dihydroxybenzene. *o*-BDOAH_2_ (0.05 g, 2.5 mmol) and 4,4'-bipyridine (0.16 g, 1.0 mmol) were dissolved in 15 ml water, and then the pH was adjusted to about 5.5 using 0.5 *M* NaOH solution with stirring. The resulting solution was stand in the atmosphere for several days. Colorless block crystals of title compound were obtained.

### Refinement

H atoms bound to C atoms were placed in calculated positions and treated as riding on their parent atoms, with C—H = 0.93 Å (aromatic C), C—H = 0.97 Å (methene C), and with *U*_iso_(H) = 1.2*U*_eq_(C). Water H atoms and carboxyl H atoms were initially located in a difference Fourier map but they were treated with O—H = 0.82 - 0.85 Å, and with *U*_iso_(H) = 1.5*U*_eq_(O). The pyridine ring of the centrosymmetric 4,4'-bipyridine molecule is rotational disordered (approximately along the N1A···C23A/N1B···C23B axis), which are splitted into two positions with free refined occupancy of 0.682 (17) and 0.318 (17), respectively.

### Crystal data

**Table:**

[Na(C_10_H_9_O_6_)(C_10_H_10_O_6_)]·0.5C_10_H_8_N_2_·H_2_O	*Z* = 2
*M**_r_* = 570.45	*F*(000) = 594
Triclinic, *P*1	*D*_x_ = 1.482 Mg m^−^^3^
Hall symbol: -P 1	Mo *K*α radiation, λ = 0.71073 Å
*a* = 8.427 (4) Å	Cell parameters from 9090 reflections
*b* = 13.135 (7) Å	θ = 3.1–27.6°
*c* = 13.411 (9) Å	µ = 0.14 mm^−^^1^
α = 111.08 (2)°	*T* = 291 K
β = 102.03 (2)°	Block, colorless
γ = 103.17 (2)°	0.24 × 0.23 × 0.21 mm
*V* = 1277.9 (13) Å^3^	

### Data collection

**Table:**

Rigaku R-AXIS RAPID diffractometer	4457 independent reflections
Radiation source: fine-focus sealed tube	3497 reflections with *I* > 2σ(*I*)
graphite	*R*_int_ = 0.022
ω scan	θ_max_ = 25.0°, θ_min_ = 3.1°
Absorption correction: multi-scan (*ABSCOR*; Higashi, 1995)	*h* = −9→10
*T*_min_ = 0.968, *T*_max_ = 0.972	*k* = −15→15
9959 measured reflections	*l* = −15→15

### Refinement

**Table:**

Refinement on *F*^2^	Primary atom site location: structure-invariant direct methods
Least-squares matrix: full	Secondary atom site location: difference Fourier map
*R*[*F*^2^ > 2σ(*F*^2^)] = 0.066	Hydrogen site location: inferred from neighbouring sites
*wR*(*F*^2^) = 0.213	H-atom parameters constrained
*S* = 1.14	*w* = 1/[σ^2^(*F*_o_^2^) + (0.1059*P*)^2^ + 1.3876*P*] where *P* = (*F*_o_^2^ + 2*F*_c_^2^)/3
4457 reflections	(Δ/σ)_max_ < 0.001
392 parameters	Δρ_max_ = 0.79 e Å^−^^3^
152 restraints	Δρ_min_ = −0.43 e Å^−^^3^

### Special details

**Table:**

Geometry. All e.s.d.'s (except the e.s.d. in the dihedral angle between two l.s. planes) are estimated using the full covariance matrix. The cell e.s.d.'s are taken into account individually in the estimation of e.s.d.'s in distances, angles and torsion angles; correlations between e.s.d.'s in cell parameters are only used when they are defined by crystal symmetry. An approximate (isotropic) treatment of cell e.s.d.'s is used for estimating e.s.d.'s involving l.s. planes.
Refinement. 1. omit -3 50 2. eqiv $4 - *x* -1, -*y*, -*z DFIX* 1.485 0.015 C23A C23A_$4 C23B C23B_$4 3. SAME N1B C21B C22B C23B C24B C25B SAME N1A C21A C22A C23A C24A C25A SIMU C21A C22A C23A C24A C25A C21B C22B C23B C24B C25B SIMU N1A N1BRefinement of *F*^2^ against ALL reflections. The weighted *R*-factor *wR* and goodness of fit *S* are based on *F*^2^, conventional *R*-factors *R* are based on *F*, with *F* set to zero for negative *F*^2^. The threshold expression of *F*^2^ > σ(*F*^2^) is used only for calculating *R*-factors(gt) *etc*. and is not relevant to the choice of reflections for refinement. *R*-factors based on *F*^2^ are statistically about twice as large as those based on *F*, and *R*- factors based on ALL data will be even larger.

### Fractional atomic coordinates and isotropic or equivalent isotropic displacement parameters (Å^2^)

**Table:**

	*x*	*y*	*z*	*U*_iso_*/*U*_eq_	Occ. (<1)
N1A	0.6188 (9)	0.2495 (3)	0.2549 (4)	0.057 (3)	0.682 (17)
C21A	0.5554 (11)	0.1402 (4)	0.2509 (3)	0.051 (2)	0.682 (17)
H21A	0.5436	0.1326	0.3156	0.062*	0.682 (17)
C22A	0.5096 (11)	0.0423 (3)	0.1503 (4)	0.0451 (19)	0.682 (17)
H22A	0.4672	−0.0309	0.1476	0.054*	0.682 (17)
C23A	0.5272 (8)	0.0536 (4)	0.0537 (3)	0.042 (3)	0.682 (17)
C24A	0.5907 (14)	0.1629 (6)	0.0577 (5)	0.050 (2)	0.682 (17)
H24A	0.6024	0.1706	−0.0069	0.060*	0.682 (17)
C25A	0.6365 (14)	0.2609 (4)	0.1583 (6)	0.061 (3)	0.682 (17)
H25A	0.6789	0.3340	0.1610	0.073*	0.682 (17)
N1B	0.591 (2)	0.2514 (7)	0.2486 (8)	0.062 (7)	0.318 (17)
C21B	0.461 (3)	0.1518 (9)	0.2270 (11)	0.067 (5)	0.318 (17)
H21B	0.4017	0.1518	0.2782	0.080*	0.318 (17)
C22B	0.420 (3)	0.0521 (7)	0.1287 (11)	0.056 (5)	0.318 (17)
H22B	0.3333	−0.0145	0.1142	0.067*	0.318 (17)
C23B	0.509 (2)	0.0521 (9)	0.0521 (7)	0.049 (8)	0.318 (17)
C24B	0.6381 (16)	0.1517 (12)	0.0738 (10)	0.036 (4)	0.318 (17)
H24B	0.6974	0.1517	0.0225	0.043*	0.318 (17)
C25B	0.6791 (16)	0.2514 (10)	0.1720 (11)	0.029 (3)	0.318 (17)
H25B	0.7658	0.3180	0.1865	0.034*	0.318 (17)
C1	1.2333 (5)	0.9344 (3)	0.3705 (3)	0.0449 (9)	
C2	1.2214 (5)	0.9812 (3)	0.4873 (3)	0.0427 (9)	
H2A	1.3347	1.0083	0.5419	0.051*	
H2B	1.1801	1.0465	0.4995	0.051*	
C3	1.1024 (5)	0.9213 (3)	0.6109 (3)	0.0393 (8)	
C4	1.1633 (5)	1.0313 (3)	0.6957 (3)	0.0496 (10)	
H4	1.2166	1.0933	0.6827	0.060*	
C5	1.1456 (6)	1.0503 (4)	0.8009 (4)	0.0616 (12)	
H5	1.1865	1.1251	0.8580	0.074*	
C6	1.0677 (6)	0.9588 (4)	0.8206 (4)	0.0633 (12)	
H6	1.0564	0.9718	0.8913	0.076*	
C7	1.0057 (6)	0.8471 (4)	0.7356 (3)	0.0537 (10)	
H7	0.9524	0.7854	0.7491	0.064*	
C8	1.0231 (5)	0.8276 (3)	0.6309 (3)	0.0404 (8)	
C9	0.8604 (5)	0.6264 (3)	0.5498 (3)	0.0432 (9)	
H9A	0.7599	0.6441	0.5650	0.052*	
H9B	0.9204	0.6126	0.6111	0.052*	
C10	0.8062 (5)	0.5200 (3)	0.4395 (3)	0.0424 (9)	
C11	1.4603 (5)	0.7310 (3)	0.4655 (3)	0.0405 (8)	
C12	1.4237 (5)	0.6162 (3)	0.3699 (3)	0.0441 (9)	
H12A	1.4988	0.6235	0.3251	0.053*	
H12B	1.4432	0.5606	0.3987	0.053*	
C13	1.1877 (5)	0.4758 (3)	0.2073 (3)	0.0453 (9)	
C14	1.2722 (7)	0.3957 (4)	0.1820 (4)	0.0633 (12)	
H14	1.3785	0.4095	0.2312	0.076*	
C15	1.1982 (8)	0.2955 (4)	0.0837 (5)	0.0784 (16)	
H15	1.2553	0.2419	0.0665	0.094*	
C16	1.0408 (7)	0.2740 (4)	0.0108 (4)	0.0755 (16)	
H16	0.9909	0.2051	−0.0544	0.091*	
C17	0.9552 (6)	0.3544 (4)	0.0336 (4)	0.0629 (13)	
H17	0.8508	0.3408	−0.0174	0.076*	
C18	1.0267 (5)	0.4546 (3)	0.1329 (3)	0.0454 (9)	
C19	0.7868 (5)	0.5188 (4)	0.0964 (3)	0.0524 (11)	
H19A	0.7130	0.4413	0.0765	0.063*	
H19B	0.7944	0.5247	0.0274	0.063*	
C20	0.7118 (5)	0.6051 (4)	0.1570 (4)	0.0558 (11)	
Na1	1.05417 (13)	0.69107 (9)	0.36791 (8)	0.0184 (3)	
O1	1.3113 (4)	1.0102 (3)	0.3415 (3)	0.0670 (9)	
H62	1.3358	1.0765	0.3903	0.100*	
O2	1.1770 (4)	0.8307 (2)	0.3072 (2)	0.0532 (7)	
O3	1.1079 (3)	0.8946 (2)	0.5032 (2)	0.0402 (6)	
O4	0.9707 (3)	0.7210 (2)	0.5416 (2)	0.0451 (6)	
O5	0.8786 (4)	0.5185 (2)	0.3693 (2)	0.0567 (8)	
O6	0.6840 (5)	0.4372 (2)	0.4313 (3)	0.0691 (10)	
H61	0.6665	0.3817	0.3714	0.104*	
O7	1.3450 (4)	0.7679 (3)	0.4858 (2)	0.0601 (8)	
O8	1.6201 (3)	0.7822 (2)	0.5237 (3)	0.0588 (8)	
O9	1.2474 (3)	0.5785 (2)	0.3023 (2)	0.0503 (7)	
O10	0.9534 (3)	0.5391 (2)	0.1663 (2)	0.0499 (7)	
O11	0.7838 (4)	0.6794 (3)	0.2531 (3)	0.0636 (8)	
O12	0.5598 (4)	0.5927 (4)	0.0985 (3)	0.0941 (14)	
H121	0.5767	0.5753	0.0368	0.141*	
O13	0.4114 (5)	0.7809 (3)	0.1738 (4)	0.0892 (12)	
H131	0.4615	0.7313	0.1724	0.134*	
H132	0.3730	0.8057	0.2284	0.134*	

### Atomic displacement parameters (Å^2^)

**Table:**

	*U*^11^	*U*^22^	*U*^33^	*U*^12^	*U*^13^	*U*^23^
N1A	0.066 (5)	0.043 (6)	0.052 (6)	0.014 (4)	0.018 (4)	0.014 (5)
C21A	0.058 (5)	0.042 (4)	0.041 (3)	0.001 (3)	0.020 (3)	0.012 (3)
C22A	0.051 (5)	0.034 (3)	0.036 (3)	−0.002 (3)	0.015 (3)	0.010 (2)
C23A	0.049 (5)	0.037 (6)	0.034 (6)	0.011 (4)	0.018 (4)	0.008 (5)
C24A	0.076 (6)	0.034 (3)	0.049 (4)	0.024 (4)	0.023 (4)	0.023 (3)
C25A	0.082 (7)	0.042 (4)	0.062 (5)	0.027 (4)	0.025 (4)	0.020 (4)
N1B	0.098 (13)	0.044 (12)	0.024 (9)	−0.010 (8)	0.029 (8)	0.008 (8)
C21B	0.097 (14)	0.044 (8)	0.052 (8)	0.013 (9)	0.034 (9)	0.014 (6)
C22B	0.061 (10)	0.044 (7)	0.047 (8)	0.001 (7)	0.020 (8)	0.012 (6)
C23B	0.061 (11)	0.040 (12)	0.043 (13)	0.005 (9)	0.014 (9)	0.023 (10)
C24B	0.045 (8)	0.034 (7)	0.036 (6)	0.021 (6)	0.010 (5)	0.020 (5)
C25B	0.044 (6)	0.020 (5)	0.030 (6)	0.012 (4)	0.009 (5)	0.020 (4)
C1	0.047 (2)	0.033 (2)	0.046 (2)	0.0057 (16)	0.0147 (17)	0.0109 (17)
C2	0.0401 (19)	0.0312 (19)	0.045 (2)	0.0077 (15)	0.0110 (16)	0.0084 (16)
C3	0.0376 (19)	0.0364 (19)	0.0345 (19)	0.0144 (15)	0.0075 (15)	0.0059 (15)
C4	0.057 (2)	0.033 (2)	0.046 (2)	0.0150 (17)	0.0147 (19)	0.0028 (17)
C5	0.075 (3)	0.045 (2)	0.038 (2)	0.016 (2)	0.015 (2)	−0.0074 (19)
C6	0.074 (3)	0.064 (3)	0.034 (2)	0.016 (2)	0.019 (2)	0.005 (2)
C7	0.063 (3)	0.054 (2)	0.040 (2)	0.019 (2)	0.0191 (19)	0.0151 (19)
C8	0.043 (2)	0.0342 (19)	0.0324 (18)	0.0142 (15)	0.0088 (15)	0.0032 (15)
C9	0.054 (2)	0.0333 (19)	0.041 (2)	0.0136 (16)	0.0191 (17)	0.0139 (16)
C10	0.052 (2)	0.034 (2)	0.042 (2)	0.0178 (17)	0.0177 (18)	0.0148 (17)
C11	0.040 (2)	0.0375 (19)	0.0386 (19)	0.0119 (16)	0.0063 (16)	0.0150 (16)
C12	0.038 (2)	0.045 (2)	0.043 (2)	0.0134 (16)	0.0134 (16)	0.0118 (17)
C13	0.053 (2)	0.039 (2)	0.0298 (18)	0.0097 (17)	0.0136 (17)	0.0017 (16)
C14	0.073 (3)	0.051 (3)	0.053 (3)	0.026 (2)	0.022 (2)	0.003 (2)
C15	0.088 (4)	0.056 (3)	0.066 (3)	0.029 (3)	0.025 (3)	−0.004 (2)
C16	0.082 (4)	0.050 (3)	0.057 (3)	0.013 (2)	0.028 (3)	−0.017 (2)
C17	0.055 (3)	0.063 (3)	0.040 (2)	−0.002 (2)	0.0141 (19)	0.003 (2)
C18	0.051 (2)	0.041 (2)	0.0325 (19)	0.0070 (17)	0.0195 (17)	0.0044 (16)
C19	0.040 (2)	0.065 (3)	0.033 (2)	0.0003 (19)	0.0008 (16)	0.0171 (19)
C20	0.033 (2)	0.078 (3)	0.049 (3)	0.010 (2)	0.0011 (18)	0.030 (2)
Na1	0.0191 (5)	0.0129 (5)	0.0135 (5)	0.0036 (4)	0.0030 (4)	−0.0026 (4)
O1	0.081 (2)	0.0409 (17)	0.063 (2)	−0.0012 (15)	0.0347 (17)	0.0113 (15)
O2	0.0685 (19)	0.0348 (15)	0.0458 (16)	0.0087 (13)	0.0225 (14)	0.0088 (13)
O3	0.0462 (14)	0.0321 (13)	0.0340 (13)	0.0111 (11)	0.0135 (11)	0.0059 (11)
O4	0.0557 (16)	0.0328 (13)	0.0371 (14)	0.0091 (11)	0.0181 (12)	0.0061 (11)
O5	0.075 (2)	0.0370 (15)	0.0499 (17)	0.0111 (14)	0.0299 (15)	0.0090 (13)
O6	0.094 (2)	0.0361 (16)	0.064 (2)	0.0022 (16)	0.0425 (18)	0.0086 (14)
O7	0.0496 (17)	0.0565 (18)	0.0506 (17)	0.0271 (14)	0.0010 (14)	−0.0001 (14)
O8	0.0388 (15)	0.0465 (16)	0.0674 (19)	0.0063 (12)	0.0076 (14)	0.0086 (14)
O9	0.0458 (15)	0.0449 (15)	0.0371 (14)	0.0177 (12)	0.0029 (12)	−0.0031 (12)
O10	0.0428 (15)	0.0551 (17)	0.0328 (14)	0.0081 (12)	0.0052 (11)	0.0069 (12)
O11	0.0488 (17)	0.064 (2)	0.063 (2)	0.0190 (15)	0.0049 (15)	0.0179 (17)
O12	0.051 (2)	0.139 (4)	0.069 (2)	0.035 (2)	0.0010 (18)	0.026 (2)
O13	0.086 (3)	0.084 (3)	0.109 (3)	0.034 (2)	0.039 (2)	0.046 (2)

### Geometric parameters (Å, °)

**Table:**

N1A—C21A	1.3900	C9—H9A	0.9700
N1A—C25A	1.3900	C9—H9B	0.9700
C21A—C22A	1.3900	C10—O5	1.221 (5)
C21A—H21A	0.9300	C10—O6	1.269 (5)
C22A—C23A	1.3900	C11—O7	1.217 (5)
C22A—H22A	0.9300	C11—O8	1.283 (5)
C23A—C24A	1.3900	C11—C12	1.499 (5)
C23A—C23A^i^	1.497 (7)	C12—O9	1.434 (5)
C24A—C25A	1.3900	C12—H12A	0.9700
C24A—H24A	0.9300	C12—H12B	0.9700
C25A—H25A	0.9300	C13—O9	1.372 (4)
N1B—C21B	1.3900	C13—C14	1.383 (6)
N1B—C25B	1.3900	C13—C18	1.407 (6)
C21B—C22B	1.3900	C14—C15	1.376 (6)
C21B—H21B	0.9300	C14—H14	0.9300
C22B—C23B	1.3900	C15—C16	1.373 (8)
C22B—H22B	0.9300	C15—H15	0.9300
C23B—C24B	1.3900	C16—C17	1.391 (7)
C23B—C23B^i^	1.516 (12)	C16—H16	0.9300
C24B—C25B	1.3900	C17—C18	1.382 (6)
C24B—H24B	0.9300	C17—H17	0.9300
C25B—H25B	0.9300	C18—O10	1.373 (5)
C1—O2	1.227 (5)	C19—O10	1.421 (5)
C1—O1	1.289 (5)	C19—C20	1.491 (7)
C1—C2	1.497 (6)	C19—H19A	0.9700
C2—O3	1.421 (5)	C19—H19B	0.9700
C2—H2A	0.9700	C20—O11	1.218 (5)
C2—H2B	0.9700	C20—O12	1.294 (5)
C3—C4	1.372 (5)	Na1—O7	2.374 (3)
C3—O3	1.373 (5)	Na1—O2	2.379 (3)
C3—C8	1.403 (6)	Na1—O11	2.407 (3)
C4—C5	1.387 (6)	Na1—O5	2.416 (3)
C4—H4	0.9300	Na1—O4	2.495 (3)
C5—C6	1.375 (7)	Na1—O3	2.501 (3)
C5—H5	0.9300	Na1—O9	2.524 (3)
C6—C7	1.387 (6)	Na1—O10	2.531 (3)
C6—H6	0.9300	O1—H62	0.8261
C7—C8	1.379 (6)	O6—H61	0.8239
C7—H7	0.9300	O12—H121	0.8280
C8—O4	1.372 (4)	O13—H131	0.8502
C9—O4	1.426 (5)	O13—H132	0.8500
C9—C10	1.513 (5)		
			
C21A—N1A—C25A	120.0	H12A—C12—H12B	108.5
C22A—C21A—N1A	120.0	O9—C13—C14	125.3 (4)
C22A—C21A—H21A	120.0	O9—C13—C18	114.6 (3)
N1A—C21A—H21A	120.0	C14—C13—C18	120.1 (4)
C21A—C22A—C23A	120.0	C15—C14—C13	119.7 (5)
C21A—C22A—H22A	120.0	C15—C14—H14	120.2
C23A—C22A—H22A	120.0	C13—C14—H14	120.2
C24A—C23A—C22A	120.0	C16—C15—C14	120.6 (5)
C24A—C23A—C23A^i^	121.5 (7)	C16—C15—H15	119.7
C22A—C23A—C23A^i^	118.5 (8)	C14—C15—H15	119.7
C23A—C24A—C25A	120.0	C15—C16—C17	120.6 (4)
C23A—C24A—H24A	120.0	C15—C16—H16	119.7
C25A—C24A—H24A	120.0	C17—C16—H16	119.7
C24A—C25A—N1A	120.0	C18—C17—C16	119.5 (4)
C24A—C25A—H25A	120.0	C18—C17—H17	120.3
N1A—C25A—H25A	120.0	C16—C17—H17	120.3
C21B—N1B—C25B	120.0	O10—C18—C17	125.0 (4)
N1B—C21B—C22B	120.0	O10—C18—C13	115.4 (3)
N1B—C21B—H21B	120.0	C17—C18—C13	119.6 (4)
C22B—C21B—H21B	120.0	O10—C19—C20	109.9 (3)
C23B—C22B—C21B	120.0	O10—C19—H19A	109.7
C23B—C22B—H22B	120.0	C20—C19—H19A	109.7
C21B—C22B—H22B	120.0	O10—C19—H19B	109.7
C24B—C23B—C22B	120.0	C20—C19—H19B	109.7
C24B—C23B—C23B^i^	113.6 (18)	H19A—C19—H19B	108.2
C22B—C23B—C23B^i^	126.1 (18)	O11—C20—O12	122.4 (5)
C23B—C24B—C25B	120.0	O11—C20—C19	123.8 (4)
C23B—C24B—H24B	120.0	O12—C20—C19	113.8 (4)
C25B—C24B—H24B	120.0	O7—Na1—O2	78.09 (12)
C24B—C25B—N1B	120.0	O7—Na1—O11	160.33 (12)
C24B—C25B—H25B	120.0	O2—Na1—O11	84.79 (12)
N1B—C25B—H25B	120.0	O7—Na1—O5	116.02 (13)
O2—C1—O1	121.8 (4)	O2—Na1—O5	162.48 (11)
O2—C1—C2	122.7 (4)	O11—Na1—O5	82.69 (12)
O1—C1—C2	115.4 (3)	O7—Na1—O4	87.39 (12)
O3—C2—C1	110.6 (3)	O2—Na1—O4	129.15 (10)
O3—C2—H2A	109.5	O11—Na1—O4	96.11 (12)
C1—C2—H2A	109.5	O5—Na1—O4	64.66 (10)
O3—C2—H2B	109.5	O7—Na1—O3	72.13 (10)
C1—C2—H2B	109.5	O2—Na1—O3	66.79 (10)
H2A—C2—H2B	108.1	O11—Na1—O3	92.31 (11)
C4—C3—O3	124.4 (4)	O5—Na1—O3	125.85 (11)
C4—C3—C8	119.8 (4)	O4—Na1—O3	62.37 (9)
O3—C3—C8	115.8 (3)	O7—Na1—O9	63.92 (10)
C3—C4—C5	120.2 (4)	O2—Na1—O9	90.25 (11)
C3—C4—H4	119.9	O11—Na1—O9	126.36 (11)
C5—C4—H4	119.9	O5—Na1—O9	87.35 (11)
C6—C5—C4	120.1 (4)	O4—Na1—O9	126.16 (10)
C6—C5—H5	120.0	O3—Na1—O9	133.82 (10)
C4—C5—H5	120.0	O7—Na1—O10	123.12 (11)
C5—C6—C7	120.2 (4)	O2—Na1—O10	88.79 (11)
C5—C6—H6	119.9	O11—Na1—O10	65.49 (11)
C7—C6—H6	119.9	O5—Na1—O10	74.88 (11)
C8—C7—C6	119.9 (4)	O4—Na1—O10	137.55 (10)
C8—C7—H7	120.0	O3—Na1—O10	148.93 (10)
C6—C7—H7	120.0	O9—Na1—O10	61.03 (9)
O4—C8—C7	125.0 (4)	C1—O1—H62	111.3
O4—C8—C3	115.3 (3)	C1—O2—Na1	121.2 (3)
C7—C8—C3	119.7 (3)	C3—O3—C2	116.6 (3)
O4—C9—C10	108.7 (3)	C3—O3—Na1	121.5 (2)
O4—C9—H9A	109.9	C2—O3—Na1	115.1 (2)
C10—C9—H9A	109.9	C8—O4—C9	117.2 (3)
O4—C9—H9B	109.9	C8—O4—Na1	122.4 (2)
C10—C9—H9B	109.9	C9—O4—Na1	120.3 (2)
H9A—C9—H9B	108.3	C10—O5—Na1	123.5 (2)
O5—C10—O6	126.2 (4)	C10—O6—H61	107.1
O5—C10—C9	120.8 (3)	C11—O7—Na1	126.1 (3)
O6—C10—C9	113.0 (3)	C13—O9—C12	117.2 (3)
O7—C11—O8	124.7 (4)	C13—O9—Na1	122.5 (2)
O7—C11—C12	121.4 (3)	C12—O9—Na1	120.0 (2)
O8—C11—C12	113.8 (3)	C18—O10—C19	117.3 (3)
O9—C12—C11	107.5 (3)	C18—O10—Na1	121.9 (2)
O9—C12—H12A	110.2	C19—O10—Na1	117.8 (2)
C11—C12—H12A	110.2	C20—O11—Na1	122.4 (3)
O9—C12—H12B	110.2	C20—O12—H121	97.1
C11—C12—H12B	110.2	H131—O13—H132	117.5

Symmetry codes: (i) −*x*+1, −*y*, −*z*.

### Hydrogen-bond geometry (Å, °)

**Table:**

*D*—H···*A*	*D*—H	H···*A*	*D*···*A*	*D*—H···*A*
O1—H62···O8^ii^	0.83	1.70	2.517 (4)	173
O6—H61···N1B	0.82	1.76	2.568 (7)	168
O6—H61···N1A	0.82	1.76	2.573 (5)	171
O13—H132···O2^iii^	0.85	2.16	2.958 (5)	156

Symmetry codes: (ii) −*x*+3, −*y*+2, −*z*+1; (iii) *x*−1, *y*, *z*.
